# AI-Generated Diet and Exercise Recommendations for Cardiovascular Health Compared to Established Cardiology Society Guidelines

**DOI:** 10.7759/cureus.90968

**Published:** 2025-08-25

**Authors:** Tagbo C Nduka, Andrew Ndakotsu, Valentine C Nriagu, Suganya Karikalan, Lukan Abdulkareem, Faith O Omede, Tamunoinemi Bob-Manuel

**Affiliations:** 1 Medicine, CHRISTUS Health/Texas A and M University, Longview, USA; 2 Internal Medicine, MedStar Georgetown University Hospital, Washington, USA; 3 Epidemiology and Public Health, East Tennessee State University, Johnson City, USA; 4 Internal Medicine, Maimonides Medical Center, Brooklyn, USA; 5 Internal Medicine, Venkataeswara Hospitals, Chennai, IND; 6 Cardiology, Advocate Illinois Masonic Medical Center, Chicago, USA; 7 Internal Medicine, North Shore Physicians Group, Mass General Brigham, Beverly, USA; 8 Interventional Cardiology, The Stern Cardiovascular Foundation, Memphis, USA

**Keywords:** artificial intelligence, cardiovascular diseases, diet, exercise, large language model

## Abstract

Background and aim: As numerous individuals turn to the internet for initial guidance, health literacy is crucial for establishing sound health practices within the broader society. Although diet and exercise are crucial for prevention, artificial intelligence (AI) models can offer accurate health information; cardiovascular disease (CVD) continues to be a significant source of morbidity and mortality. The increasing application of AI in healthcare necessitates a thorough evaluation of the efficacy of large language models (LLMs) in providing dependable health recommendations. This study aimed to assess the appropriateness, biases, and clinical relevance of diet and exercise recommendations produced by four prominent language models (ChatGPT {San Francisco, CA: OpenAI}, Claude AI {San Francisco, CA: Anthropic}, DeepSeek AI {Hangzhou, China: DeepSeek}, Google Gemini {Google LLC: Mountain View, CA}) in relation to established cardiovascular disease association guidelines from the American Heart Association/American College of Cardiology (AHA/ACC) and the European Society of Cardiology (ESC).

Methods: A cross-sectional study was conducted using 15 standardized questions (five on physical activity, 10 on diet) evaluated by a primary care physician and a cardiology fellow. A cardiologist reviewed discrepancies in the evaluations by the two examiners; in such instances, the final grade was established by the median of the grades assigned by all three examiners. Based on compliance with AHA/ACC and ESC guidelines, responses were rated as appropriate, appropriate but insufficient, partially inappropriate, or entirely inappropriate.

Results: Ninety percent of responses from ChatGPT, Claude AI, and DeepSeek AI met established cardiovascular health standards, indicating superior performance among the language models. All five recommendations for physical activity were deemed appropriate. Google Gemini had a performance level of 80%, while 90% of the outcomes from the three LLMs were suitable for nutritional guidance. Particularly concerning carbohydrate and added sugar intake, all models struggled to provide precise quantitative guidance. Exercise recommendations indicated a slight preference for AHA/ACC guidelines.

Conclusions: While LLMs demonstrate potential for accessible health information sources, they cannot replace expert medical advice. This study highlights the need for continued medical professional interpretation and tailored healthcare guidance. Future advances should concentrate on raising the specificity of health recommendations and guaranteeing a fairer interpretation of international guidelines.

## Introduction

For many years, cardiovascular disease (CVD) has been the primary cause of morbidity and death. Primarily due to poor diet and inactivity - both of which readily predispose individuals to obesity (currently an epidemic), elevated blood pressure, hyperlipidemia, and type 2 diabetes, all significant risk factors for heart disease such as coronary artery disease, heart failure, rhythm disorders, and stroke - the burden of cardiovascular disease continues to increase faster than our capacity to prevent it [1,2]. Diet and exercise are crucial for prevention and disease management, with cardiologists emphasizing an evidence-based lifestyle.

Numerous patients inquire about lifestyle alterations, specifically regarding dietary choices and exercise regimens, to prevent the onset of cardiovascular illnesses. Formally, these questions are directed to the primary care doctors or cardiologists. For many decades, the internet has served as a source of adjunctive health information for patients, oftentimes simplifying what would otherwise be complex medical literature. However, with the increasing visibility of social media applications, such as X (formerly known as Twitter {San Francisco, CA: X Corp.}), Instagram (Menlo Park, CA: Meta), TikTok (Beijing, China: ByteDance), etc., as well as ease of access to artificial intelligence (AI) software, the likelihood of patients relying on information gleaned from these sources is already upon us [3].

Cardiovascular disease organizations around the globe have established websites focused on patient-relevant subjects to enhance validated information and patient education concerning food and exercise. This development signifies a wider transition towards digital self-education as individuals utilize internet platforms to enhance conventional medical consultations. In response to this transition, prominent CVD organizations globally, like the American Heart Association/American College of Cardiology (AHA/ACC) and the European Society of Cardiology (ESC), have developed websites focused on delivering patient-centered information on essential subjects, including nutrition, physical activity, and disease prevention. These efforts seek to provide public access to verified medical information, enabling individuals to make educated health decisions. Moreover, novel digital instruments for patient education are continually being developed, providing interactive and tailored methods for understanding cardiovascular health [4].

Although few comparative studies have assessed the alignment of AI-generated lifestyle recommendations with existing cardiovascular guidelines from the AHA/ACC and ESC, prior research has explored AI applications in the management of metabolic disorders. Despite insufficient investigation into their accuracy and reliability, large language models (LLMs) are increasingly utilized in healthcare. This raises concerns over health misinformation and negative consequences linked to off-label use. Despite these concerns, the widespread use of AI-driven chatbots has required the establishment of quality standards and the specification of constraints for patient use. The increasing reliance on AI-generated medical information underscores the essential need to evaluate its reliability and address its limitations for patient safety [4-6].

This study aimed to assess the appropriateness, comprehensibility, and clinical relevance of chatbot-generated recommendations to evaluate their potential as reliable adjunct tools in preventive cardiology and to ensure alignment with established evidence-based guidelines from leading cardiovascular organizations. Additionally, it seeks to identify possible instances of misinformation and determine whether these AI tools align with evidence-based recommendations provided by reputable cardiovascular organizations. By analyzing the accuracy and reliability of AI-driven medical advice, this study will contribute to the ongoing discussion about the role of LLMs in healthcare and help inform the development of guidelines for their responsible use in patient education.

## Materials and methods

This is a cross-sectional study with non-human participants. Recommendations were compiled from diverse cardiovascular disease association guidelines worldwide, such as the AHA/ACC and ESC, and later converted into questions [7,8]. A total of 15 questions were posed, with five related to physical activity and 10 focused on diet (Tables 1, 2). This study mainly focused on diet and exercise due to their comparative clarity and practicality.

**Table 1:** The questions on exercise and responses from language models. Some of the responses have been condensed to fit into the table. Nonetheless, all essential points have been preserved in the table. HIIT: high intensity interval training

Questions on exercise	ChatGPT	Claude AI	DeepSeek AI	Google Gemini
What is considered a low/light-intensity exercise for cardiovascular disease prevention?	150-300 min per week (2.5-5 h) of activities like walking, stretching, or light cycling, or 30-60 min per day, five days a week, or 10-15 min spread throughout the day also count. Focus on gentle activities that slightly elevate your heart rate while allowing conversation.	Light-intensity exercises like walking, swimming, cycling, yoga, gardening, stretching, dancing, and resistance training can help prevent cardiovascular disease by raising heart rate, allowing conversation, and sustaining comfortably. Consistency is key.	Low/light-intensity exercise, such as walking, cycling, swimming, yoga, or gardening, can prevent cardiovascular disease, improve circulation, lower blood pressure, and reduce stress. Start slowly and gradually increase intensity.	Low-intensity exercise, such as walking, bicycling, swimming, rowing, yoga, water aerobics, tai chi, light resistance training, golf, and yard work, promotes cardiovascular disease prevention, reduces fatigue, improves mood, reduces falls, injuries, and improves sleep quality, minimizing joint impact.
How many times can I exercise in a week to prevent cardiovascular diseases?	150-300 min per week (2.5-5 h) of exercise or five to seven days per week, with 30-60 min per session, or shorter sessions of 10-15 min, multiple times a day, are effective. Consistency across the week is key for maximum benefit.	Cardiovascular disease prevention requires 150-150 min of moderate-intensity exercise or 75-150 min of vigorous-intensity exercise per week, with 300-150 min for enhanced benefits, including rest days and aerobic and strength training.	To prevent cardiovascular diseases, engage in 150-75 min of moderate-intensity exercise per week, including strength training twice a week and low-intensity activities. Start gradually, consult a healthcare provider, and maintain consistency.	For optimal cardiovascular health, aim for at least 150 min of moderate-intensity or 75 min of vigorous-intensity aerobic exercise per week, spread across multiple days, combined with strength training at least two days a week. Regular, consistent activity, tailored to your fitness level and in consultation with your doctor, is crucial for disease prevention.
What is considered a moderate-intensity exercise for cardiovascular disease prevention?	150-300 min per week (2.5-5 h). Examples include brisk walking, cycling on flat terrain, or dancing. Break this into 30-60 min per day, five days a week, or shorter bouts of 10-15 min spread throughout the day. Activities should elevate your heart rate but still allow you to talk.	Moderate-intensity exercise, such as walking, cycling, and swimming, increases heart rate and breathing, allowing for short conversations, and is indicated by quick breathing, sweating, and warmth.	Moderate-intensity exercise, including walking, cycling, swimming, and sports, is essential for cardiovascular disease prevention. It improves cholesterol, lowers blood pressure, boosts endothelial function, reduces inflammation, and aids weight management. Recommendations include 150-300 min of aerobic exercise per week.	Moderate-intensity exercise, such as walking, cycling, and swimming, increases heart rate and breathing rate while allowing conversation. Aim for 150 min per week for cardiovascular health benefits. Intensity varies based on fitness level.
What is considered a high/vigorous-intensity exercise for cardiovascular disease prevention?	High/vigorous-intensity exercise for cardiovascular disease prevention requires 75-150 min per week (1.25-2.5 h). This can be divided into 15-30 min per day, five days a week. Activities include running, swimming laps, or cycling uphill. These exercises should significantly raise your heart rate, making talking difficult during the activity.	Vigorous-intensity exercise, such as jogging, swimming, tennis, cycling, and aerobic dancing, can increase heart rate and breathing rate, making it difficult to maintain conversation.	High/vigorous-intensity exercise, including running, swimming, cycling, HIIT, and competitive sports, can improve cardiovascular health, lower blood pressure, and manage weight, but should be combined with moderate-intensity strength training.	Vigorous-intensity exercise, such as running, HIIT, fast swimming, and cycling, significantly increases heart rate and breathing, strengthening the heart and improving its efficiency.
How many minutes of exercise are considered the minimum needed in a week to prevent cardiovascular diseases?	The minimum exercise needed to prevent cardiovascular diseases is 150 min per week of moderate-intensity activity or 75 min per week of vigorous-intensity activity. This can be spread over five days, with sessions of 30 min (moderate) or 15 min (vigorous). Consistency is crucial for heart health benefits.	The recommended weekly exercise for cardiovascular disease prevention is 150 min of moderate-intensity or 75 min of vigorous-intensity exercise, with shorter sessions being better.	Regular aerobic activity, including moderate and vigorous workouts, is recommended for cardiovascular disease prevention. Older adults should focus on muscle-strengthening activities and balance training. High-risk individuals should also incorporate functional exercises.	The recommended weekly exercise for cardiovascular disease prevention is 150 min of moderate-intensity aerobic activity, 75 min of vigorous aerobic activity, or a combination of both, spread throughout the week. Adults should aim for 300 min or more moderate-intensity exercise for greater health benefits.

**Table 2:** The questions on diets and responses from language models. Some of the responses have been condensed to fit into the table. Nonetheless, all essential points have been preserved in the table. LDL: low-density lipoprotein; HDL: high-density lipoprotein; HIIT: high-intensity interval training

Questions on diet	ChatGPT	Claude AI	DeepSeek AI	Google Gemini
What is a heart-healthy diet?	A heart-healthy diet, incorporating fruits, vegetables, whole grains, lean proteins, healthy fats, low-fat dairy, portion control, water, alcohol moderation, and balanced eating patterns, supports cardiovascular health.	A heart-healthy diet includes fruits, vegetables, whole grains, lean proteins, healthy fats, low-fat dairy, herbs, and spices while limiting saturated fats, trans fats, sugars, sodium, refined carbohydrates, and alcohol.	A heart-healthy diet, incorporating fruits, vegetables, whole grains, healthy fats, lean proteins, low sodium, and sugars, promotes overall wellness and reduces cardiovascular risks by avoiding trans fats, high-sodium snacks, and ultra-processed foods.	A heart-healthy diet reduces cardiovascular disease risk by incorporating fruits, vegetables, whole grains, and lean proteins, limiting saturated fats, trans fats, cholesterol, sodium, and added sugars, and promoting fiber and antioxidants.
What amount of carbohydrates is good for my heart?	For heart health, carbohydrates should make up about 45-65% of your daily calories, focusing on complex carbs from whole grains, vegetables, fruits, and legumes. Avoid refined carbs like white bread, pastries, and sugary drinks, which can spike blood sugar and increase heart risk. Aim for 25-38 g of fiber daily, as it supports heart health by reducing cholesterol. The exact amount depends on your calorie needs and health goals; typically, 225-325 g daily for a 2,000-calorie diet.	Focus on complex carbohydrates from whole grains, legumes, vegetables, and fruits for 45-65% of daily calories. Limit refined carbohydrates and added sugars to 25-36 g per day.	Focus on quality, prioritize fiber-rich, complex carbs, and avoid refined carbs and added sugars. Key principles for heart-healthy carbs include 25-35 g of fiber daily, limiting added sugars to <10% of daily calories, choosing "slow" carbs like whole grains, legumes, fruits, and non-starchy vegetables, and avoiding "fast" carbs like refined grains and sugary drinks. Adjust carb intake based on health status, such as diabetes or prediabetes, metabolic syndrome or obesity, and active individuals. Low-carb diets are not typically recommended for long-term heart health. Practical tips include reading labels, avoiding added sugars or enriched flour, pairing carbs with protein/fat, and cooking at home.	The recommended daily carbohydrate intake for heart health is 45% to 65%, resulting in 225-325 g of carbohydrates. To maintain heart health, choose complex carbohydrates like whole grains, vegetables, fruits, and legumes, limit simple carbohydrates, aim for fiber-rich foods, and balance carbohydrates with protein and healthy fats. Individual factors like age, activity level, and health conditions may influence optimal carbohydrate intake.
What are saturated fats?	Saturated fats are fats that are solid at room temperature and are primarily found in animal products like butter, cheese, fatty meats, and certain plant oils (e.g., coconut and palm oil). They can raise LDL ("bad") cholesterol levels, increasing the risk of heart disease.	Saturated fats are solid dietary fats with a rigid structure due to their saturated carbon atoms with hydrogen atoms. Common sources include animal products, tropical oils, and processed foods.	Saturated fats, found in animal and plant-based foods, can increase LDL cholesterol, heart disease risk, and inflammation. To reduce saturated fat intake, opt for lean proteins, switch dairy to low-fat or fat-free options, cook smartly, and read labels.	Saturated fats, found in animal-based foods and tropical oils, can increase LDL cholesterol levels and, risk of heart disease and stroke. Health organizations recommend limiting saturated fat intake to 5-6% of daily calories.
What is a Mediterranean diet?	The Mediterranean diet, a heart-healthy plan based on Mediterranean foods, promotes vegetables, fruits, whole grains, legumes, nuts, olive oil, moderate fish, moderate dairy, and moderate wine consumption.	The Mediterranean diet, a combination of vegetables, fruits, whole grains, and minimally processed foods, offers health benefits like reduced heart disease risk, improved blood sugar control, and longer life expectancy.	The Mediterranean diet is a balanced diet rich in nutrients, healthy fats, and antioxidants, incorporating plant-based foods, whole grains, legumes, olive oil, fish, moderate protein, poultry, dairy, eggs, and moderate red meat.	The Mediterranean diet, originating from Southern Italy and Greece, is a plant-based, low-processed, whole food diet high in fiber, heart-healthy fats, and antioxidants, which may reduce cardiovascular disease risk, type 2 diabetes risk, improve cognitive function, and weight loss.
Can I eat fish or meat? Which one is good for my heart health?	Consume fish and lean meats in moderation for a heart-healthy diet, focusing on omega-3 fatty acids and limiting processed and red meats to reduce heart disease risk.	Fish and meat can be part of a healthy diet, with general recommendations being around 2-3 times per week for fatty fish like salmon, mackerel, and sardines. Lean cuts like chicken breast, turkey, and pork tenderloin are best. Limit red meat and poultry to 2-3 times per week.	Consume fatty fish 2-3 times per week for omega-3s and lean cuts, limiting red and processed meats. Maintain a balanced diet with fruits, vegetables, whole grains, and healthy fats.	Fish, particularly oily ones like salmon, are beneficial for heart health due to omega-3 fatty acids, while red meat, especially processed types, can increase heart disease risk.
What quantity of salt is good for my heart?	For heart health, limit salt (sodium) intake to less than 2,300 mg per day (about 1 teaspoon) and aim for 1,500 mg per day if you have high blood pressure or are at risk for heart disease. Excess sodium can raise blood pressure, increasing cardiovascular risk. Reduce processed and packaged foods, which are major sources of hidden salt. Use herbs, spices, and natural flavors as healthier alternatives to enhance meals.	To maintain heart health, it's crucial to monitor and limit salt intake. The ideal daily limit is 1,500 mg, with higher-risk groups like high blood pressure, diabetes, kidney disease, and African Americans needing closer to 1,500 mg. To reduce salt intake, read food labels, cook meals at home, use herbs and spices, rinse canned vegetables and beans, and be cautious with processed and restaurant foods.	For heart health, most adults should limit sodium intake to no more than 2,300 mg per day, with an ideal target of 1,500 mg per day for those with high blood pressure or heart disease. To reduce salt intake, avoid processed foods, cook at home, and use herbs and spices for flavor.	For heart health, limit sodium intake to no more than 2,300 mg/day (general recommendation). Ideally, 1,500 mg/day (especially for those with high blood pressure). Processed foods are a major source of sodium, so always read labels.
What dairy products can I eat, and which dairy product is good for my heart?	For heart health, choose low-fat or fat-free dairy products like skim milk, low-fat yogurt, and reduced-fat cheese. These options provide calcium and protein without the saturated fat found in full-fat versions. Unsweetened plant-based alternatives like almond or soy milk are also good choices. Limit high-fat options like cream, butter, and full-fat cheeses to reduce cholesterol and saturated fat intake.	Heart-healthy dairy products include low-fat or fat-free milk, plain low-fat yogurt, kefir, low-fat cottage cheese, and part-skim ricotta. These provide beneficial nutrients like calcium, potassium, and vitamin D without excessive saturated fat. The American Heart Association recommends limiting full-fat dairy products, such as cheese, cream, ice cream, butter, and whole milk, to reduce saturated fat intake that may raise cholesterol levels.​​	Low-fat dairy options like yogurt, skim milk, cottage cheese, and kefir are recommended for heart health. Moderate consumption of full-fat dairy, processed cheeses, and heavy cream is advised. Plant-based alternatives include unsweetened milk, coconut yogurt, and nut-based cheeses.	Greek yogurt, cheese, and milk. Limit intake to two cups daily, opt for low-fat options, and use cheese as a condiment. For dairy-free alternatives, consider unsweetened almond milk, soy milk, or oatmeal.
What is a DASH diet?	The Dietary Approaches to Stop Hypertension (DASH) diet is designed to lower blood pressure and improve heart health. It emphasizes fruits, vegetables, whole grains, lean proteins, and low-fat dairy while limiting salt, red meat, processed foods, and added sugars. The diet encourages nutrient-rich foods high in potassium, calcium, magnesium, and fiber. DASH is proven to reduce hypertension and promote overall cardiovascular health.	The DASH diet is a balanced eating plan designed to treat or prevent high blood pressure. It focuses on reducing sodium intake, consuming whole foods, and limiting processed foods. The diet offers health benefits such as lower blood pressure, heart disease risk, weight management, diabetes risk, and certain cancer risks. It is often paired with regular exercise, stress management, and adequate sleep.	The DASH diet is a heart-healthy eating plan focused on lowering blood pressure and improving health. It emphasizes fruits, vegetables, whole grains, and lean proteins; low-fat dairy and healthy fats; low sodium (≤2,300 mg/day, or 1,500 mg for better results); and limited sweets. Rich in nutrients and fiber, it’s effective for heart health and blood pressure management.	The DASH diet is a healthy eating plan designed to lower blood pressure. It emphasizes: whole foods: fruits, vegetables, whole grains. Lean protein: fish, poultry, beans. Low-fat dairy. Limits: saturated fat, sodium, added sugars. It promotes overall health and reduces heart disease risk.
What type of oil is associated with low cardiovascular disease risk?	Oils rich in unsaturated fats, like extra virgin olive oil, are associated with a lower risk of cardiovascular disease. Olive oil, a staple of the Mediterranean diet, contains heart-healthy monounsaturated fats and antioxidants that reduce inflammation and improve cholesterol levels. Other healthy options include avocado oil, canola oil, and oils high in omega-3 fatty acids like flaxseed oil. Avoid trans fats and limit the saturated fats found in coconut and palm oils.	Regular consumption of olive oil, along with avocado oil and omega-3 fatty acids, has been linked to reduced cardiovascular disease risk, heart attacks, strokes, and mortality rates, according to multiple studies.	Monounsaturated and polyunsaturated fats in olive oil, canola, avocado, and flaxseed oil reduce LDL and increase HDL while avoiding saturated and trans fats. Use in moderation in a balanced diet.	Olive oil, particularly extra virgin, is strongly associated with lower cardiovascular disease risk due to its high content of monounsaturated fatty acids and antioxidants, which help lower "bad" cholesterol and protect blood vessels. Studies consistently demonstrate that regular consumption of olive oil, especially when replacing less healthy fats, contributes to improved heart health.
What amount of added sugar is good for my heart?	Limit added sugar intake to 6-9 g per day for women and 36 g per day for men for heart health. Avoid sugary drinks, desserts, and processed foods, and focus on natural fruits.	The American Heart Association advises limiting added sugar intake to 6 teaspoons daily for women and 9 teaspoons for men for optimal heart health, instead opting for natural sugars from fruits, vegetables, and dairy.	Limit added sugars to 6-25 g per day for women and 9 g per day for men for heart health. Avoid sugary drinks, processed foods, and use natural sweeteners sparingly.	Consuming less added sugar, especially from sugary drinks, is crucial for heart health, as high intake increases the risk of heart disease, and a low-sugar diet is essential for optimal health.

To choose the questions, we conducted a comprehensive literature assessment of the AHA/ACC and ESC guidelines, which enabled us to identify the essential areas of attention vital for preserving cardiovascular health. To guarantee that the selected questions were pertinent and actionable, we classified them into two primary categories as follows: activity and diet. We incorporated questions regarding varying intensity levels (low, moderate, and high), exercise frequency, and minimum time of physical activity, all of which are essential elements of an efficient exercise plan (Table 1). In the nutritional segment, we emphasized essential components of cardiovascular health, including heart-healthy dietary patterns, varieties of carbs and lipids, as well as specific regimens, such as the Mediterranean and Dietary Approaches to Stop Hypertension (DASH) diets. We also examined significant factors, such as sugar consumption and meat intake (Table 2). By integrating ideas from established cardiology guidelines, we developed a set of 15 questions that emphasize essential elements of cardiovascular health and provide a substantive comparison between AI-generated recommendations and expert guidelines. This guaranteed that our investigation was thorough and pertinent, consequently enhancing the comprehension of the significance LLMs play in heart health recommendations.

Four notable language models, specifically ChatGPT (GPT-4, March 2023 version), Claude AI (Claude 2, September 2023 version), DeepSeek AI (DeepSeek-LLM 67B, January 2024 version), and Google Gemini (Pro version, December 2023), were queried with the questions. ChatGPT is a prominent big language model developed by OpenAI, designed for conversational interaction and providing responses that mimic human-like dialogue and knowledge across various topics. Claude AI, created by Anthropic, emphasizes safety and alignment in artificial intelligence, highlighting ethical principles and enabling user-friendly interactions in conversational settings. DeepSeek AI specializes in deep learning applications, particularly in the effective search and retrieval of information from large datasets, enhancing data-driven decision-making. Furthermore, Google Gemini aims to integrate AI capabilities into many goods to provide contextually pertinent responses and supportive functionalities. All the previously listed models showed competence in understanding texts and generating human-like responses. These questions were formulated in comprehensible English, appropriate for an individual with average educational qualifications within the general public seeking health information.

All responses provided by each LLM to the posed questions were evaluated as appropriate, appropriate but insufficient, partially inappropriate, or entirely inappropriate, based on their compliance with the guideline requirements. A primary care physician and a cardiology fellow independently evaluated and rated the responses. Discrepancies in the evaluations by the two examiners were subjected to a third review conducted by a cardiologist; in such instances, the final grade was established by the median of the grades assigned by all three examiners. This process ensured a balanced and unbiased assessment of the AI recommendations. No patient data were documented in this study; hence, institutional review board permission was not required.

## Results

Quantitative assessment

**Figure 1:**
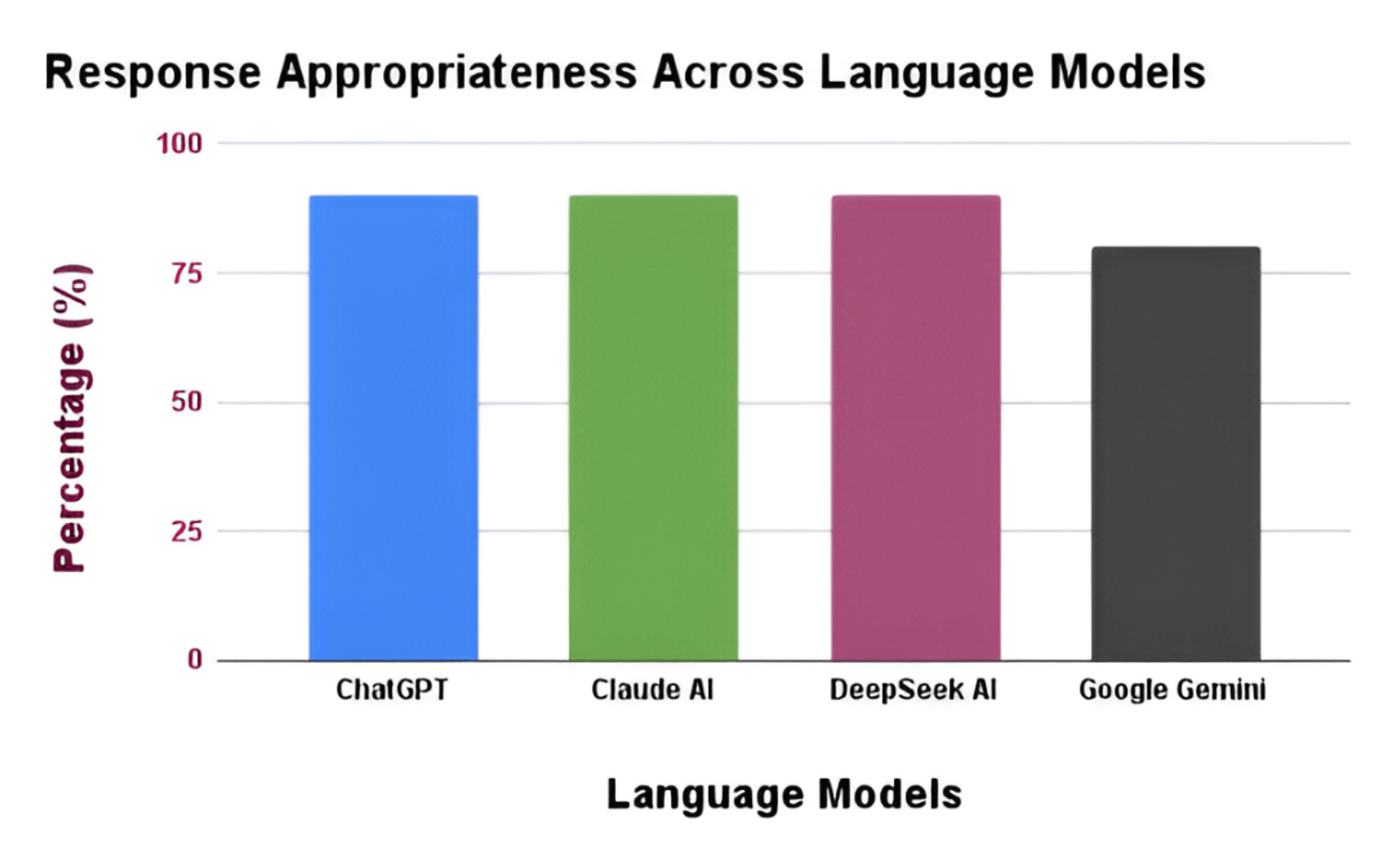
A bar chart depicting the performance of language models in terms of appropriateness. ChatGPT (San Francisco, CA: OpenAI), Claude AI (San Francisco, CA: Anthropic), DeepSeek AI (Hangzhou, China: DeepSeek), Google Gemini (Google LLC: Mountain View, CA)

Of the 15 questions presented, five responses concerning physical activities generated by the four LLMs were all considered appropriate. None of the responses were deemed to be appropriate but insufficient, partially inappropriate, or entirely inappropriate for the questions posed for physical activities. Of the 10 responses about diet, 90% (9/10) generated by ChatGPT (San Francisco, CA: OpenAI), Claude AI (San Francisco, CA: Anthropic), and DeepSeek AI (Hangzhou, China: DeepSeek) were found to be appropriate, whereas one question was seen as appropriate but insufficient. In the assessment of Google Gemini (Google LLC: Mountain View, CA), 80% (8/10) of the responses were classified as appropriate, while two responses were regarded as appropriate but insufficient (Figure 1). No responses were either partially or entirely inappropriate for the diet-related responses. The responses from DeepSeek AI and Google Gemini were overly lengthy, requiring a subsequent prompt of "summarize the answer" for improved clarity. ChatGPT and Claude AI produced a higher proportion of responses consistent with the required guidelines compared to DeepSeek AI and Google Gemini. While most responses generated were considered to be unbiased, several responses generated by the LLMs seemed to preferentially endorse the recommendations from the AHA/ACC over those from the ESC.

Qualitative assessment

ChatGPT

All responses generated by ChatGPT were considered appropriate. The response regarding the carbohydrate recommendation in the diet area, specifically asked as “what amount of carbohydrate is good for my heart,” was deemed appropriate but insufficient. This is due to ChatGPT's inability to deliver a precise response deemed comprehensive or closely aligned with the recommendations from either AHA/ACC or ESC. The ChatGPT response emphasized broad guidelines by advocating for entire diets beneficial for cardiovascular health, such as fruits and vegetables. The response did not address the optimal carbohydrate intake for cardiovascular health as outlined in the guidelines.

Claude AI

Similar to ChatGPT, all responses generated by Claude AI were considered appropriate. The question regarding the carbohydrate recommendation was posed as, "What amount of carbohydrate is good for my heart?" The response to this question was deemed appropriate but insufficient, as it failed to deliver a comprehensive and precise description of the carbohydrate requirements for cardiovascular health according to the AHA/ACC and ESC guidelines.

DeepSeek AI

All responses produced by DeepSeek AI were deemed appropriate. Similar to ChatGPT and Claude AI, this chatbot provided informative content but did not entirely conform to the particular carbohydrate guidelines set forth by the AHA/ACC and ESC regarding the question, “What amount of carbohydrate is good for my heart?” DeepSeek underscored the significance of consuming high-quality carbs and fiber. It did not explicitly address the ramifications of both elevated and reduced carbohydrate percentages linked to increased mortality, as outlined in the guidelines. Therefore, the response to the question “What amount of carbohydrate is beneficial for my heart?” Was graded as appropriate but insufficient.

Google Gemini

All responses provided by Google Gemini were appropriate. Nevertheless, two responses from the dietary part were deemed appropriate but insufficient. The question “What amount of carbohydrates is good for my heart?” Similar to ChatGPT, Claude AI, and DeepSeek AI, Google Gemini did not furnish a thorough answer that aligns with the AHA/ACC and ESC guidelines. Instead, it emphasized the selection of complex carbohydrates, such as whole grains, while also incorporating vegetables, fruits, legumes, proteins, and healthy fats, the majority of which are not classified as carbohydrates. The AHA/ACC guidelines classify beneficial carbohydrate sources for heart health as whole grains, such as brown rice, oats, and some types of popcorn. The ESC recommendations emphasized the spectrum of carbohydrate consumption and overall energy intake. Consequently, this response was deemed appropriate but insufficient. Google Gemini did not deliver a satisfactory answer to the question, “What quantity of added sugar is good for my heart?” The response highlighted the significance of restricting added sugar but failed to provide the particular percentage recommendations outlined by the AHA/ACC and ESC guidelines. Both AHA/ACC and ESC recommendations recommend capping added sugar consumption at less than 10% of total daily energy intake; hence, this reaction was deemed appropriate but insufficient.

Bias assessment

**Figure 2:**
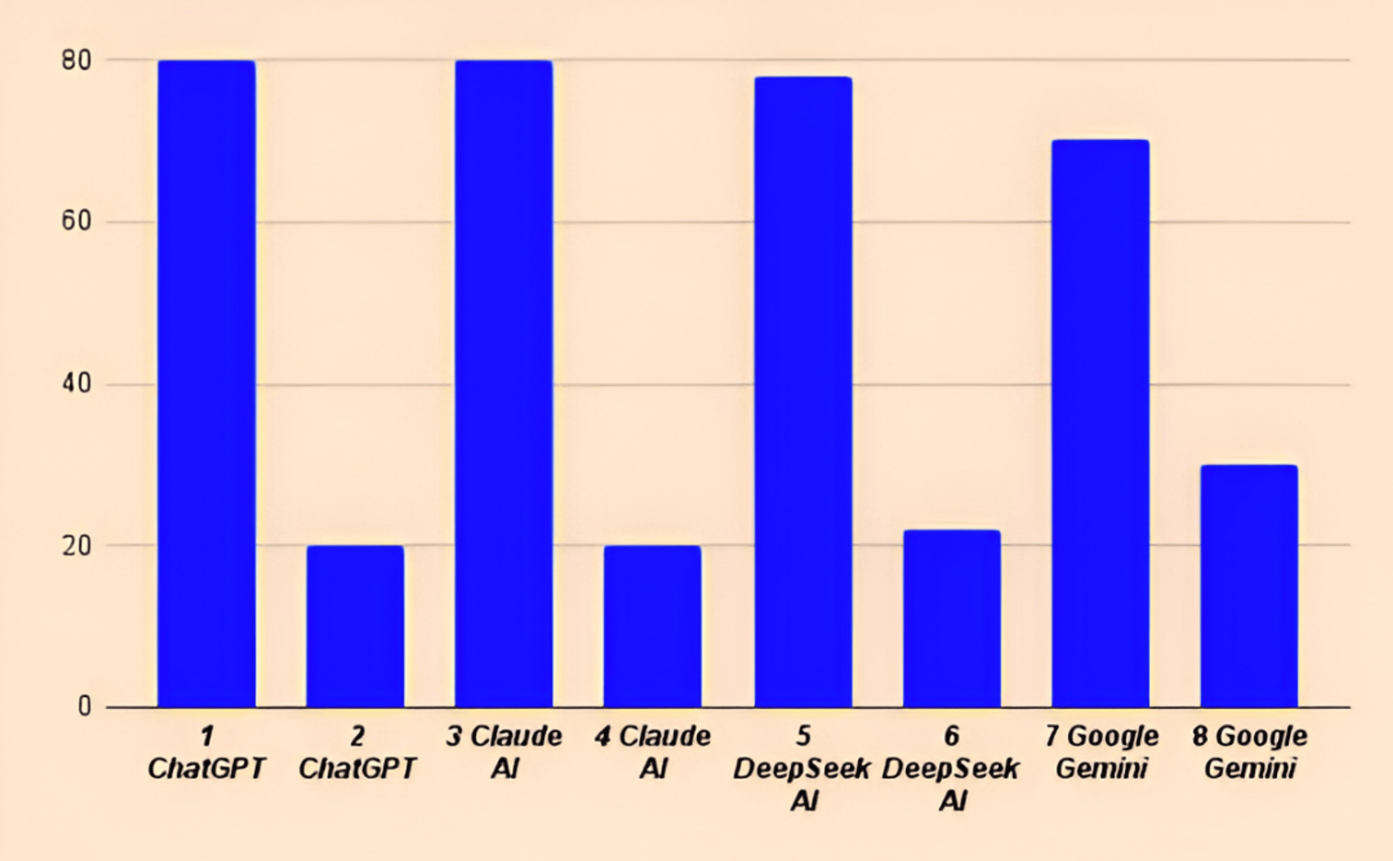
Alignment of large language models with cardiovascular guidelines. This figure presents a comparative analysis of multiple large language models’ performance adherence to cardiovascular guidelines from prominent organizations, including the American Heart Association/American College of Cardiology (AHA/ACC) and the European Society of Cardiology (ESC). Comparative performance metrics - the bar graph illustrates the alignment performance of eight language model-guideline combinations. (1) ChatGPT with AHA/ACC guidelines, (2) ChatGPT with ESC guidelines, (3) Claude AI with AHA/ACC guidelines, (4) Claude AI with ESC guidelines, (5) DeepSeek AI with AHA/ACC guidelines, (6) DeepSeek AI with ESC guidelines, (7) Google Gemini with AHA/ACC guidelines, and (8) Google Gemini with ESC guidelines. ChatGPT (San Francisco, CA: OpenAI), Claude AI (San Francisco, CA: Anthropic), DeepSeek AI (Hangzhou, China: DeepSeek), Google Gemini (Google LLC: Mountain View, CA)

The majority of the responses generated by the AI systems seemed to exhibit a greater leaning towards the AHA/ACC guidelines in comparison to the ESC guidelines (Figure 2). The first question in the exercise/physical activity section specifically asked, “What is considered a low/light exercise for cardiovascular disease prevention?” While the AHA/ACC and ESC guidelines emphasized activities, such as slow walking, cooking, and light housework. Generative language models incorporated these recommendations while also encompassing additional activities, including stretching, light cycling, gentle swimming, yoga/tai chi, light gardening, gentle dancing, light resistance training, golf, rowing, and water aerobics. In response to question number 2 in the exercise section, regarding the frequency of weekly activity necessary to prevent cardiovascular issues, LLMs had a more significant bias towards the AHA/ACC recommendations compared to the ESC guidelines. No substantial biases were detected in the dietary section. Some of the responses have been condensed to fit into the tables. Nonetheless, all essential points have been preserved in the tables.

## Discussion

This study critically investigates the capacity of four well-known language models to generate recommendations for cardiovascular health. Although various studies have looked at artificial intelligence (AI) for metabolic syndrome and diabetes treatment, these studies often focus on general outcomes without evaluating alignment to guideline-specific metrics, such as carbohydrate thresholds or exercise frequency recommendations [6]. This study assessed and compared exercise and nutritional responses given by four main language models. A primary care physician and a cardiology fellow assessed the responses; any discrepancies between the two reviewers were reviewed by a cardiologist, providing the final grade. The results reveal consistent performance among all three large language models, with 90% of the responses of ChatGPT, Claude AI, and DeepSeek AI fulfilling established cardiovascular health criteria for both physical activity and dietary recommendations.

Of the 15 questions presented for responses, five were formulated to assess physical activity recommendations from the joint AHA/ACC and ESC guidelines. Considering that significant disparities and potentially biased discriminatory outputs have been identified in content related to essential elements of exercise recommendations, which may be excessive for certain patients, the four LLMs delivered appropriate responses to the five questions posed [9,10]. Nevertheless, they were primarily observed to include supplementary recommendations, such as stretching, light cycling, gentle swimming, yoga/tai chi, light gardening, gentle dancing, light resistance training, golf, rowing, and water aerobics, which were absent from the cardiovascular disease association guidelines. This indicates that elderly patients ought to heed the instructions of these LLMs with caution.

In contrast to the exercise recommendations, the questions regarding dietary recommendations exhibited certain limitations, as three LLMs (ChatGPT, Claude AI, and DeepSeek AI) delivered appropriate responses to nine out of 10 questions posed. Meanwhile, Google Gemini, with a slightly lower rate of 80% for appropriate responses, underscores minor deficiencies in offering comprehensive nutritional guidance. The responses to questions regarding carbohydrate and added sugar consumption significantly failed to meet the precise quantitative criteria outlined by AHA/ACC and ESC guidelines. Current literature emphasizes that AI can enhance, but not replace, the critical role of human experts in delivering personalized, culturally attuned, and clinically informed nutritional interventions; thus, it remains imperative for primary care physicians to persist in educating their patients regarding dietary recommendations that can aid in the prevention of cardiovascular disease [1,11,12].

This study identified the limited ability of AI-generated health recommendations to provide quantitative guidance in health advice as a significant challenge. The findings from this study's methodology indicate that Google Gemini had inferior performance compared to three AI chatbots, suggesting that responses from AI language models cannot be generalized. This underscores the importance of motivating patients to pursue professional help for their health requirements, as a previous study by Annor et al. assessed the efficacy of two language models, Microsoft Copilot (Redmond, WA: Microsoft Corp.) and Google Gemini, in providing weight loss recommendations, revealing that Google Gemini underperformed compared to Microsoft Copilot [13].

It is noteworthy that the four LLMs specifically responded to question number 2 in the physical activity section, which asked, “How many times can I exercise in a week to prevent cardiovascular diseases?” The responses produced by the AI system demonstrated a stronger inclination for the recommendations of the AHA/ACC relative to those offered by the ESC. This compelling discovery may arise from the inherent biases of the AI-powered tools or their developers. It is crucial to meticulously assess the recommendations of these LLMs to reduce biased responses. As previously mentioned, the conversational AI systems enhanced baseline recommendations by integrating a wider array of activities, indicating a propensity to offer more complete rather than strictly conforming advice. This method, although possibly advantageous, prompts issues regarding the accurate interpretation of established protocols.

Despite the potential of language models to reply to medical inquiries more swiftly, they encounter challenges related to context awareness and quality assurance. Compliance with cultural rules may be ambiguous, and the precision of information fluctuates [14]. Zhou et al. observed that while ChatGPT possesses potential in medicine, it needs training on medical databases, which may lead to inconsistent responses [15]. A study by Sarraju et al. emphasized the necessity for AI to be more interactive to facilitate effective patient education and communication [16]. This observation was corroborated in our research, where language models, such as DeepSeek AI and Google Gemini, were requested to summarize their responses for enhanced clarity and readability, particularly concerning cardiovascular prevention recommendations. It is also crucial to note that language models must recalibrate their method of conveying information for persons who have not attended university [17]. Information should be accessible to all those with mobile phones who wish to review available advice or suggestions before consulting their physicians. It is important to repeat that AI can only enhance, not replace, primary care physicians, dieticians, and preventive cardiologists.

The application of artificial intelligence in clinical practice offers opportunities for improved cardiovascular health management, despite ongoing ethical concerns and potential impacts on doctor-patient relationships. Effective implementation necessitates the closure of knowledge gaps via medical education initiatives, robust training data derived from clinical research, and accessible AI platforms that enhance clinical proficiency. This symbiotic collaboration enables physicians to leverage the computational power of artificial intelligence while maintaining their human rapport with patients [18,19].

This study demonstrates the significant potential of language models as readily accessible sources of health information. The strong relevance suggests that individuals seeking preventative cardiovascular health guidance regarding diet and exercise can significantly benefit from these models as initial resources. The results underscore the essential requirement for competent medical interpretation and individualized assistance. Future advancements in artificial intelligence health recommendation systems should emphasize enhancing quantitative accuracy in nutritional guidance, formulating more equitable interpretations of international guidelines, and expanding the ability to provide nuanced, specific health recommendations.

While this study provides valuable analysis, some limitations must be acknowledged as follows: it was conducted with a limited panel of medical specialists, assessed only four language models, and addressed merely 15 questions. Future research should augment the number of questions and models examined, using more precise quantitative assessment criteria, and explore the practical significance of health advice generated by artificial intelligence. AI language models are continually evolving, and it is essential to acknowledge that, over time, particularly in comparison to the period when this research was conducted, they will become increasingly intricate and comprehensive.

## Conclusions

This study offers important new perspectives on the use of big language models, including ChatGPT, Claude AI, DeepSeek AI, and Google Gemini, in offering cardiovascular society suggestions on exercise and diets, as well as how these recommendations may be further included in healthcare practice. Our findings indicated that expert medical counsel cannot be substituted by language models, notwithstanding their ability to provide readily accessible and fundamentally appropriate health information. Further research may be necessary to fully understand the capabilities of these language models.
